# Comparison Between Environmental DNA Metabarcoding and Traditional Survey Method to Identify Community Composition and Assembly of Stream Fish

**DOI:** 10.1002/ece3.70627

**Published:** 2024-11-25

**Authors:** Ziyu Liu, Cong Hu, Wenhui You, Shuxin Li, Yongsheng Wu, Yangyang Liang, Ling Chu, Yunzhi Yan, Chen Zhang

**Affiliations:** ^1^ School of Ecology and Environment Anhui Normal University Wuhu China; ^2^ Anhui Province Key Laboratory of Aquaculture & Stock Enhancement Fishery Institute of Anhui Academy of Agricultural Sciences Hefei China; ^3^ Anhui Normal University Provincial Key Laboratory of Biotic Environment and Ecological Safety in Anhui Wuhu China; ^4^ Collaborative Innovation Center of Recovery and Reconstruction of Degraded Ecosystem in Wanjiang Basin Co‐Founded by Anhui Province and Ministry of Education Anhui Normal University Wuhu China

**Keywords:** community assembly, functional diversity, null model, subtropical river

## Abstract

Environmental DNA (eDNA) metabarcoding has been widely used in freshwater systems, contributing to the advancements in the monitoring of fish diversity and community species composition. Nevertheless, the accuracy and reliability of eDNA metabarcoding in assessing functional structures and revealing the mechanisms underlying fish community assembly remain unclear. In this study, we combined a traditional survey method (electrofishing) and eDNA metabarcoding to conduct fish stock monitoring in the upper reaches of the Huishui stream. We assessed taxonomic and functional structures, as well as community assembly mechanisms, during dry and wet seasons. The results revealed that, compared with electrofishing surveys, eDNA metabarcoding detected a greater number of species and higher functional richness in both seasons. Despite significant differences in fish taxonomic composition between the seasons, both eDNA and traditional methods indicated that environmental filtering dominated the process of fish community assembly in both dry and wet seasons. We showed that eDNA metabarcoding is comparable to the electrofishing method in monitoring the community composition of stream fish and can accurately and reliably determine fish community assembly mechanisms. Combining functional traits and eDNA is a robust approach for monitoring stream fish community compared to taxonomic uncertainty.

## Graphical abstract

A comparison of morphology and eDNA‐based analysis was performed. eDNA metabarcoding detected a greater number of species and higher functional richness compared with electrofishing. eDNA metabarcoding can infer fish community assembly mechanisms in stream fish communities.

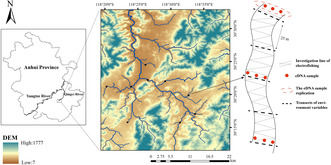

## 1 Introduction

Fish have always played a central role in providing and maintaining ecosystem services in aquatic environments due to their high biodiversity and abundance. They mediate nutrient fluxes and influence biophysical habitats (Holmlund and Hammer 1999; Pelicice et al. 2023). The diversity and distribution patterns of fish communities serve as crucial indicators reflecting the status of aquatic ecosystems and are essential components of aquatic ecosystem management (Geist 2011). However, fish diversity faces global threats due to disturbances such as climate change, biological invasions, and overfishing (Villéger et al. 2017; Su et al. 2021). Efficient monitoring of fish diversity and understanding the assembly mechanisms of fish communities are paramount tasks for the conservation of fish resources and diversity (Olden et al. 2010; Maire et al. 2013).

In recent decades, traditional fish survey methods, such as gillnets, cage traps, and electrofishing, have been the primary techniques for investigating fish diversity (Shaw et al. 2016). These methods entail professionals identifying captured individuals based on morphology, a process that is both time‐ and labor‐intensive and potentially harmful to fish populations and aquatic ecosystems (Zhang et al. 2020). In addition, accurately detecting fish with different ecological habits and rare species is challenging due to inherent biases in each fishing gear, which may result in an underestimation of fish diversity (Radinger et al. 2019; Piggott et al. 2021). Recently, eDNA metabarcoding, which identifies species based on gene sequence fragments released by organisms in the water environment, has rapidly developed in aquatic ecosystems (Wang et al. 2021; Yao et al. 2022; Mathon et al. 2023). Due to its high accuracy and sensitivity, eDNA metabarcoding is now widely used in investigations of fish populations, diversity, and community structure (Doi et al. 2023; Sahu et al. 2023; Nakagawa et al. 2018).

Understanding how environmental filtering and spatial processes jointly affect community assembly is crucial for fish conservation (Ohira et al. 2015; Tikhonov et al. 2020). Strong environmental filtering causes fish to occur only in suitable habitats, and their functional traits may be clustered (Fitzgerald et al. 2017; Camara et al. 2024). When spatial processes (e.g., dispersal) dominate, fish may arrive at diverse local communities and cause trait divergence (Zhang et al. 2021). Methods for distinguishing the relative influence of these two processes on community assembly have developed rapidly in the past decade. For instance, null models and hierarchical modeling of species communities (HMSC) can be used to determine the dominant processes in fish communities based on functional traits to guide conservation strategies (Mori et al. 2015; Montanyès, Weigel, and Lindegren 2023). With accurate species trait databases, eDNA metabarcoding can effectively detect the functional diversity and trait composition of fish communities (Marques et al. 2021; Condachou et al. 2023). Recently, some studies have shown great potential for incorporating eDNA into the inference of ecological processes in fish communities, such as determining the main driving processes of river fish community assembly at the watershed level (Gu et al. 2023; Shi et al. 2023; Li et al. 2024).

However, due to specific limitations of traditional and eDNA survey methods, such as primer and gear types, the results of these two methods in fish monitoring are not always completely congruent. This incongruence may lead to changes in the inference of fish community assembly (Keck et al. 2022). For example, Czeglédi et al. (2021) reported that compared with traditional survey methods, such as electrofishing and gillnetting, eDNA metabarcoding may underestimate the relative significance of spatial factors in fish community assembly. Moreover, differences in species richness resulting from distinct survey methods may change the functional structure of communities, leading to significant deviations in the understanding of fish community assembly and conservation strategies (Marques et al. 2021; Villéger et al. 2017). Nevertheless, few studies (Czeglédi et al. 2021) have integrated traditional and eDNA metabarcoding methods to indicate fish community assembly mechanisms, and the accuracy of eDNA‐based fish community assembly is unclear (Dal Pont et al. 2021; Hallam et al. 2021; Jerde 2021; Pont et al. 2021; Zhang et al. 2024).

Stream fish are predominantly specialized species adapted to lotic environments, playing an important role in maintaining stream ecosystems with simple habitat structures and exogenous nutrition (Ferreira et al. 2023). Because of the high specialization and narrow distribution ranges, stream fish are particularly susceptible to climate change and human disturbance (Buisson et al. 2013; Walker et al. 2020). The application of eDNA metabarcoding in stream ecosystems is more convincing than in other aquatic ecosystems (e.g., lakes) due to directional water flow, especially in the upper reaches of streams (Nakagawa et al. 2018; Penaluna et al. 2023). Typically, due to the lotic environment and spatial isolation, the functional traits of fish in the upper reaches of streams converge. and fish communities are predominantly influenced by environmental filtering (Troia and Gido 2015; Henriques‐Silva et al. 2019). Upstream fish communities may exhibit clear trait assembly patterns, which can be efficiently and accurately captured by eDNA (Condachou et al. 2023). Hence, the upper reaches of streams provide an ideal scenario for inferring the fish community assembly based on eDNA and are necessary to integrate species diversity and functional traits in the studies of fish community assembly (Benone et al. 2020).

Notably, eDNA metabarcoding signals can be affected by hydrological conditions and fish reproductive cycles, causing considerable seasonal variations (Milhau et al. 2021; Curtis et al. 2021). Therefore, cross‐seasonal studies are required to determine the reliability of using eDNA metabarcoding on stream fish communities. In this study, by integrating a traditional survey method (electrofishing) with eDNA metabarcoding, we performed a cross‐seasonal investigation of the fish community in the upper reaches of the Huishui stream. Integrating the results of the two methods with fish functional traits, we used the null models and the HMSC framework to evaluate fish community assembly across seasons. We aimed to evaluate the accuracy and reliability of eDNA metabarcoding in reflecting stream fish community structure and assembly mechanisms across seasons.

## 2 Materials and Methods

### 2.1 Study Area and Sampling Sites

**FIGURE 1:**
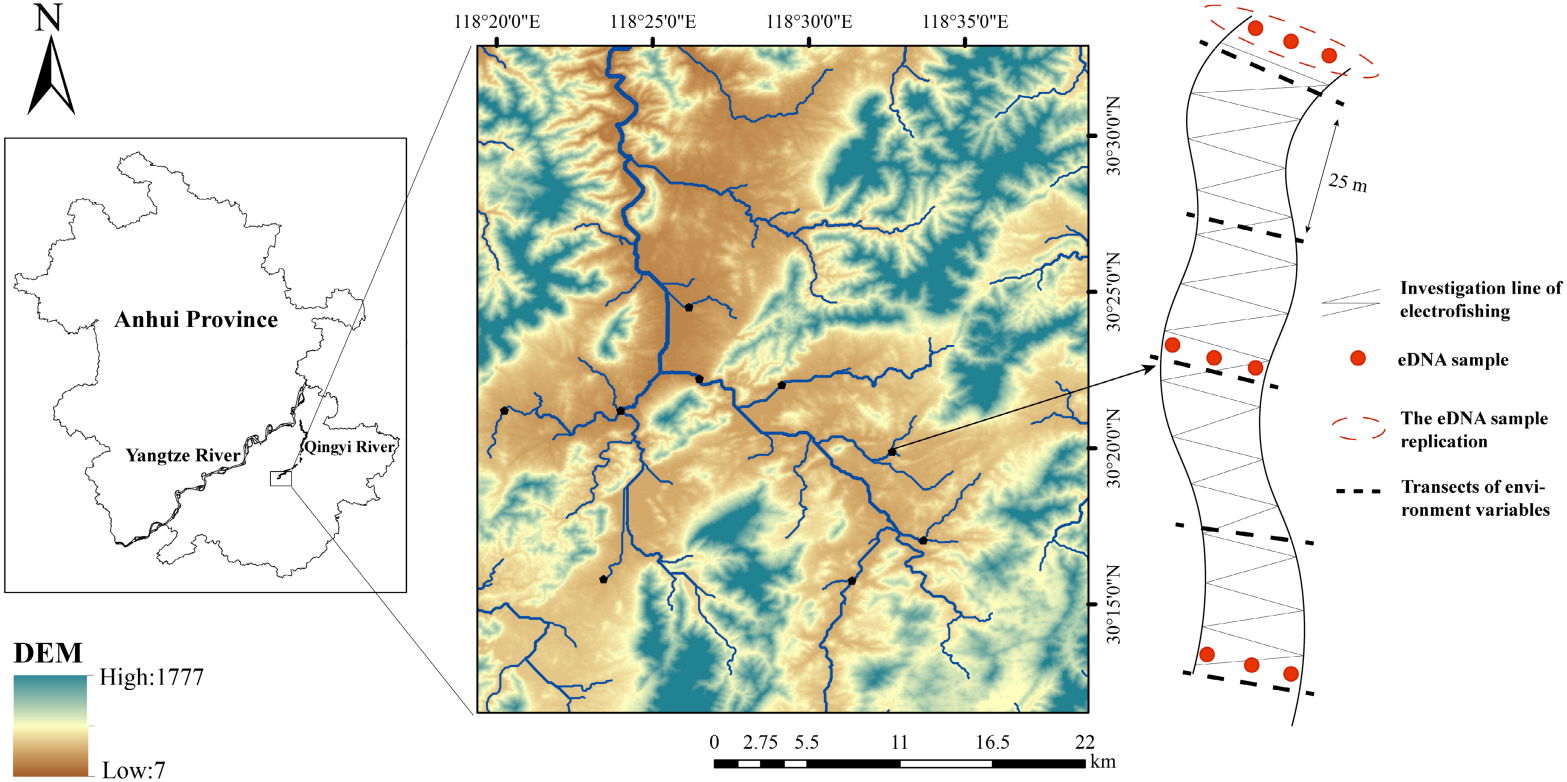
Location of the sampling areas (Huishui stream) and distribution of the sampling sites.

The Qingyi River is the largest tributary of the lower reaches of the Yangtze River, starting from the northern Huangshan Mountain in Anhui Province, China. The Huishui stream is situated in the upper reaches of the Qingyi River basin. The average width of the Huishui stream is 38.9 m, the river slope ratio is 5.1%, and the average annual flow is 4.49 m^3^/s, which is characterized as a perennial mountain stream. Influenced by the subtropical monsoon climate, the Huishui stream experiences high temperatures and rainfall during the wet seasons (March–August) and lower temperatures with minimal rainfall during the dry seasons (October–January). In this study, we included nine sampling sites within the mainstream and tributaries of the upper reaches of the Huishui stream (Figure 1). Specifically, we conducted fish investigations at each site in an approximately 100 m long stream section. These sites are situated within a fishery reserve; therefore, the fish experience minimal human disturbance. Following the guidelines and regulations set forth by the Department of Agriculture and Rural Affairs of Anhui Province (2022219), we performed simultaneous traditional fish investigations and eDNA sample collection at these sites in May and October 2023, respectively.

### 2.2 Water Sample Collection and Fish Sampling

#### 2.2.1 eDNA Collection and Processing

To reduce external contamination, water sample collection was initiated as the first step. We cleaned all sampling bottles with 10% bleach before water collection, and operators changed to fresh sterilized gloves for each stage of the sampling process (Pilliod et al. 2013). First, water samples were obtained 5 m upstream of the designated sampling reaches. Composite samples were obtained to offer a comprehensive survey of fish species across various stream habitats. Specifically, in wadable sections of the stream, we collected water samples from different microhabitats such as riffles and pools. Furthermore, water samples were collected from the left, middle, and right sections of the mainstream. Equal volumes of water samples were obtained from each microhabitat and mixed into a 2 L water sample. Then, two replicates of the 2 L water sample were collected at the middle and end of the investigated stream section, respectively. Each site collected three replicate samples, totaling nine samples and 6 L of water for fish eDNA analysis. At each site, a blank control was collected to assess potential contamination by exogenous DNA. eDNA in the water samples was then collected onto 0.45 μm mixed cellulose filter membranes (Jinteng, Tianjin) using vacuum filtration within 8 h. Before and after collecting DNA for each sample, the filtration equipment was sterilized to avoid contamination across samples (Balasingham et al. 2018). The filter membranes were transferred into 10 mL centrifuge tubes and stored at −80°C until DNA extraction.

Total DNA extraction from each filter membrane was conducted using the PowerWater DNA Isolation Kits (Qiagen, China). For amplification of the eDNA samples, the primers Tele02_F (5′‐AAACTCGTGCCAGCCACC‐3′) and Tele02_R (5′‐GGGTATCTAATCCCAGTTTG‐3′) were used (Taberlet et al. 2018). These primers target the 12S rRNA region of the mitochondrial genome (Taberlet et al. 2012). The PCR amplification system is referenced by Zhang et al. (2020). Contamination during the PCR amplification process was simultaneously monitored. The blank controls were processed alongside the samples using the same PCR protocol to monitor contamination during sampling and PCR amplification. The PCR products underwent 2% agarose gel electrophoresis for detection, followed by purification and recovery. No bands were detected in the blank controls. Finally, the samples underwent sequencing on the Illumina MiSeq platform by Lingen Biotechnology Co. Ltd. (Shanghai, China). The original sequences underwent filtration to remove low‐quality sequences using Trimmomatic v.0.36 (Bolger, Lohse, and Usadel 2014), and paired reads were combined into sequences utilizing FLASH v.1.2.11 (Magoč and Salzberg 2011). The samples were distinguished based on the barcodes and primer regions at both ends of the sequences, and the sequence orientation was adjusted using QIIME 2 v.2023.2 (Bolyen et al. 2019). The reference sequences from the GOLD database (https://gold.jgi.doe.gov/) was combined with Usearch software (Edgar 2010), using a denovo and reference‐based approach to remove chimeras. The high‐quality sequences were obtained using Usearch v.10, with a similarity threshold of ≥ 97% (Zhang et al. 2022). The sequence exhibiting the highest abundance was selected as the representative sequence for each operational taxonomic unit (OTU). Representative sequences were preliminary compared with the NCBI GenBank database by the Blastn tool, under the criteria of similarity ≥ 97%, coverage ≥ 0.9, e ≤ −10^−5^ (Zou et al. 2020). We built a 12S rRNA database based on muscle samples from previously captured fish individuals (Yan et al. 2013; Li et al. 2023). Subsequently, further taxonomic assignment was based on the self‐built libraries. In the case of a discrepancy between the comparison results of the public database and the self‐built database and the sequence similarity was both ≥ 97%, the matching result of the self‐built database was given priority. Based on the aforementioned process, the fish species annotation results were finally obtained.

#### 2.2.2 Fish Sampling and Functional Traits

We used a backpack electrofishing unit (CWB–2000P, China) for traditional fish investigation in the sampling sites. Following a zigzag line, we investigated 100 m along the length of the stream within 40 min at each site. The collected fish individuals were identified, counted, and weighed (to the nearest g) in the field. We collected 10 specimens of each species for functional traits measurement, and live fish were released back into the stream. All fish were included if less than 10 were collected. Among the collected specimens, live fish were anesthetized first using 60 mg/L tricaine methanesulfonate (MS‐222, Sigma, USA) and then placed to death in a formalin solution. Fish that could not be identified through morphological characteristics were transported to the laboratory for molecular identification. For each species, we extracted tissue DNA from fin samples to establish self‐built libraries.

To ascertain the correlation between the functional traits of fish and their habitat characteristic, 11 morphological characteristics associated with feeding, mobility, and habitat use were measured. For fish species not collected in this investigation, we obtained their trait data by measuring historical specimens or based on publicly published articles. We then calculated seven functional traits based on them (Winemiller, Fitzgerald, and Pianka 2015) (Table S1).

### 2.3 Environmental Variables

At each sampling site, we measured and recorded various physicochemical variables. The elevation, longitude, and latitude of each sampling site were recorded using a GPS measuring instrument (Magellan, USA). We used portable YSI equipment (YSI Incorporated, USA) to measure the local habitat variables, which included dissolved oxygen (DO, mg/L), conductivity (CO, μs/cm), pH, and water temperature (WT, °C). Water current velocity (CV, m/s) and water depth (WD, m) were evaluated using a portable current meter (FP11, USA), whereas water width was determined using a rangefinder. Adhering to the methodology outlined by Barbour et al. (1999), we categorized the stream substrate into the following five types based on particle size: boulders (> 256 mm), cobbles (64–256 mm), pebbles (32–64 mm), gravel (2–32 mm), and silt and clay (< 2 mm). The habitat variables were measured along five transects across the stream section at each site (Li et al. 2023).

### 2.4 Data Analysis

Before all analyses, the traditional fish abundance (number of individuals) and eDNA data (abundance of OTU) were log (*x* + 1) transformed to mitigate the impact of abundant species or OTUs.

Venn analysis was conducted to compare the outcomes of the two methods, aiding in the evaluation of the efficacy of eDNA metabarcoding for species monitoring. The VennDiagram package 1.7.3 in R 4.4.0 was used for Venn analysis (Chen and Boutros 2011; R Core Team 2023).

To evaluate the dissimilarity in fish community composition across seasons, nonmetric multidimensional scaling (NMDS) analysis was performed based on Bray–Curtis dissimilarity matrices of fish abundance and OTUs for each season. Subsequently, the analysis of similarities (ANOSIM) was employed to detect variations in fish community structure between the dry and wet seasons. NMDS and ANOSIM analyses were performed using the Vegan package 2.6.6 in R (Oksanen et al. 2015).

To analyze the functional composition of fish communities during both the dry and wet seasons, we constructed a multidimensional functional space based on fish functional traits (Villéger et al. 2017). This method calculated trait‐based distances between species pairs and created multidimensional functional spaces using community and trait matrices. We used the mFD package 1.0.7 to construct the functional space of fish communities in the dry and wet seasons and assessed seasonal differences in functional composition based on functional richness (Magneville et al. 2022). Then, we ran the null model based on functional richness (FRic), functional evenness (FEve), and functional divergence (FDiv) to determine the assembly of fish communities between the two seasons (Spasojevic and Suding 2012). The species pool was assumed to represent the fish community surveyed by both traditional and eDNA methods during the dry and wet seasons. We ran the null model based on the matrix‐swap algorithm, which randomly sampled species occurrence frequencies (Gotelli 2000). Simulations were run 999 times, and standard effect size (SES) was calculated for each functional diversity index and each season as:

$$\left({\text{mean}}_{\text{observed}}-{\text{mean}}_{\text{simulated}}\right)/{\mathrm{SD}}_{\text{simulated}}$$

where ${\text{mean}}_{\text{observed}}$ was the mean observed functional diversity index; ${\text{mean}}_{\text{simulated}}$ and ${\mathrm{SD}}_{\text{simulated}}$ were the mean functional diversity index and the standard deviation of the simulated null model, respectively. The one‐tailed permutation test (*p* < 0.05) of SES values was used to test the significance of the difference from null expectations (Cooke, Bates, and Eigenbrod 2019). Null models were performed using the mFD and the picante packages 1.8.2 in R (Magneville et al. 2022; Kembel et al. 2010). To evaluate the differences between traditional data and OTU data in inferring community assembly mechanisms, we also employed the HMSC framework. Combining taxa and functional traits, the HMSC framework serves as a prevalent tool for analyzing the relative effects of spatial and environmental factors on biological communities, offering insights into community assembly mechanism (Ovaskainen et al. 2017). Bayesian inference and Markov Chain Monte Carlo (MCMC) were used by HMSC models to determine the response patterns of each fish species and trait to fixed effects (i.e., environmental variables) and random effects (i.e., spatial factors). In this study, fixed effects included eight environmental variables, whereas geographic coordinates represented spatial random effects. We performed two MCMC chains for 1,500,000 iterations (with 500 iterations and 2000 posterior samples in each chain) and eliminated the first‐third of iterations for each chain to increase result confidence (Burgazzi et al. 2020). Model performance was evaluated based on the area under the curve (Oberdorff et al. 2011), which assesses the discriminatory ability of a species distribution model (Montanyès, Weigel, and Lindegren 2023). The AUC values of 0.5 indicated poor model performance, 0.7–0.8 was considered acceptable, 0.8–0.9 was considered excellent, and > 0.9 was considered outstanding (Mandrekar 2010). The HMSC analyses were performed using the HMSC package in R (Tikhonov et al. 2020).

## 3 Results

**FIGURE 2:**
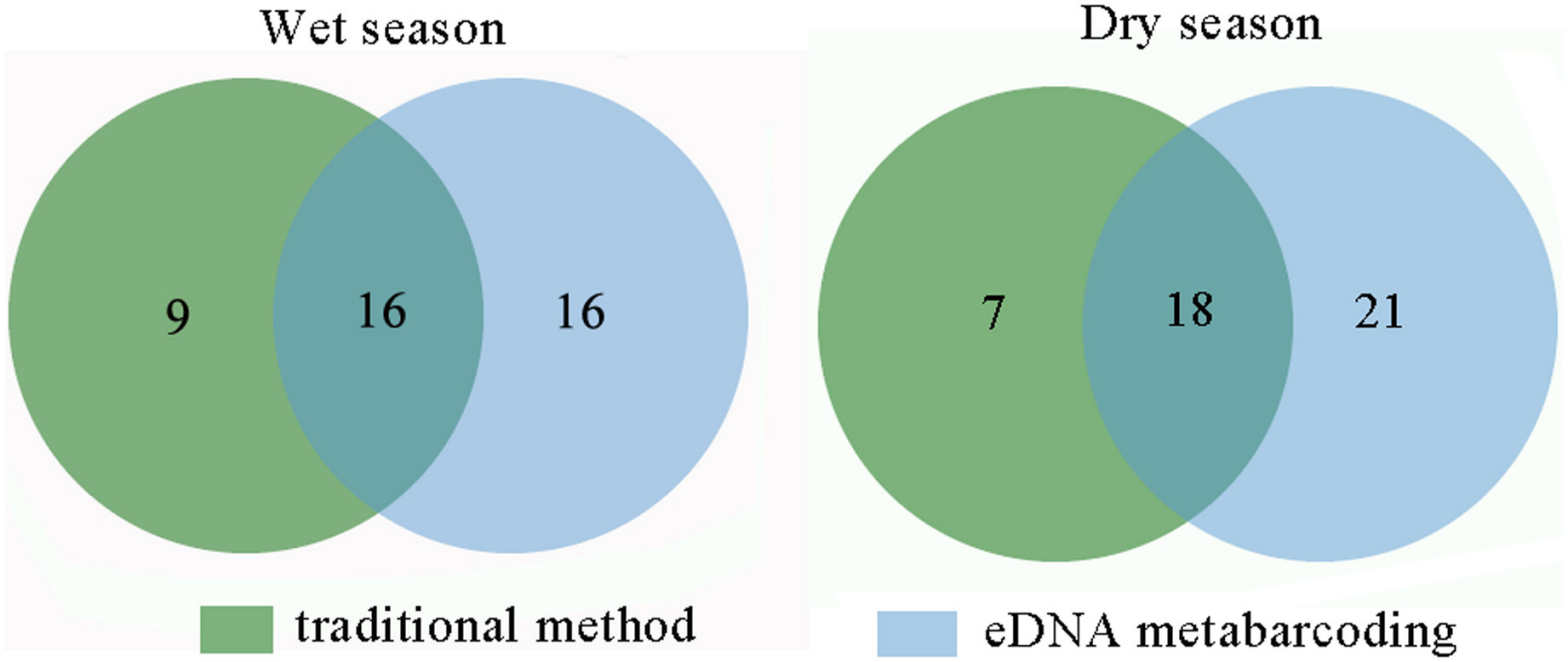
Differences in species composition monitored by the two methods during the wet season and the dry season.

Using the traditional fish survey method, 530 individuals representing 25 species in 8 families were collected in the dry season, whereas 538 individuals belonging to 25 species in 8 families were collected in the wet season. Through eDNA metabarcoding, we obtained 4, 999, 069 filtered reads clustered into 6, 274 OTUs in the wet season and 4,722,923 filtered reads clustered into 8649 OTUs in the dry season. By comparing the OTUs with public databases (NCBI) and our self‐built 12S rRNA databases, we identified 42 fish species: 32 during the wet season and 39 during the dry season. Cypriniformes species constituted the majority of species monitored using eDNA metabarcoding in both seasons (81.25% in the wet season and 67.5% in the dry season), followed by Perciformes (12.5% in the wet season and 17.5% in the dry season). Similar results were also detected using the traditional monitoring method, with Cypriniformes representing 80% in the wet season and 72% in the dry season, and Perciformes accounting for 12% in the wet season and 16% in the dry season. Using the traditional method, 34 fish species were captured, whereas 42 fish species were identified based on eDNA metabarcoding. The species coverage rates were 64% in the wet season and 72% in the dry season. In the wet season, 16 species were detected by the eDNA method, namely *Chanodichthys mongolicus* , *Opsariichthys evolans*, and *Channa maculate*. Conversely, only nine species were detected by the traditional survey method, namely *Rhinogobius cliffordpopei* , *Huigobio chenhsienensis* , and *Acheilognathus chankaensis* (Figure 2). Similarly, in the dry season, only 21 fish species were detected by eDNA metabarcoding, whereas only seven fish species were detected by the traditional survey method (Figure 2). In addition, invasive species, such as *Lepomis macrochirus* , *Oreochromis niloticus* , and *Micropterus salmoides* , were only detected by the eDNA method and never found in the Huishui stream.

**FIGURE 3:**
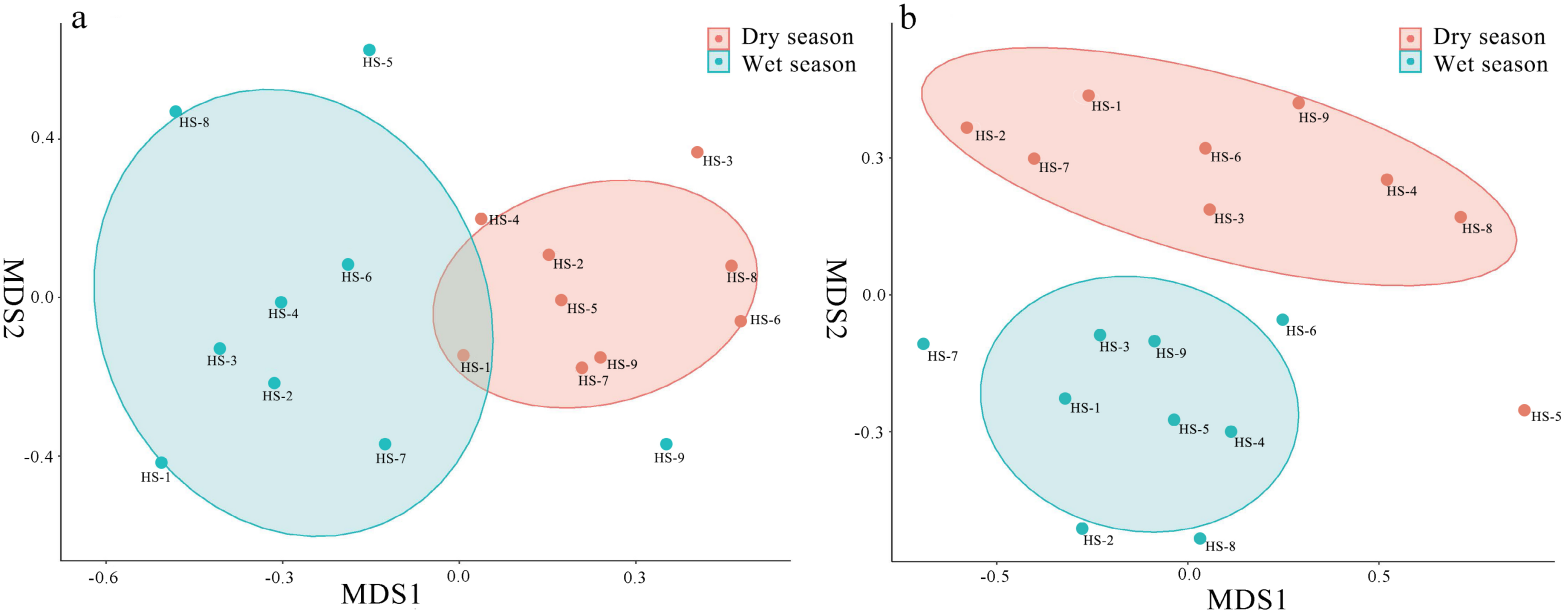
Variations in fish community composition across seasons based on eDNA metabarcoding (a) and the traditional method (b).

Fish community structures based on the two survey methods revealed similar seasonal patterns (Figure 3). Results of NMDS and ANOSIM showed that fish community structures were significantly different across seasons based on traditional fish data (*p* < 0.01, *R* = 0.445) and eDNA sequences (*p* < 0.01, *R* = 0.404).

**FIGURE 4:**
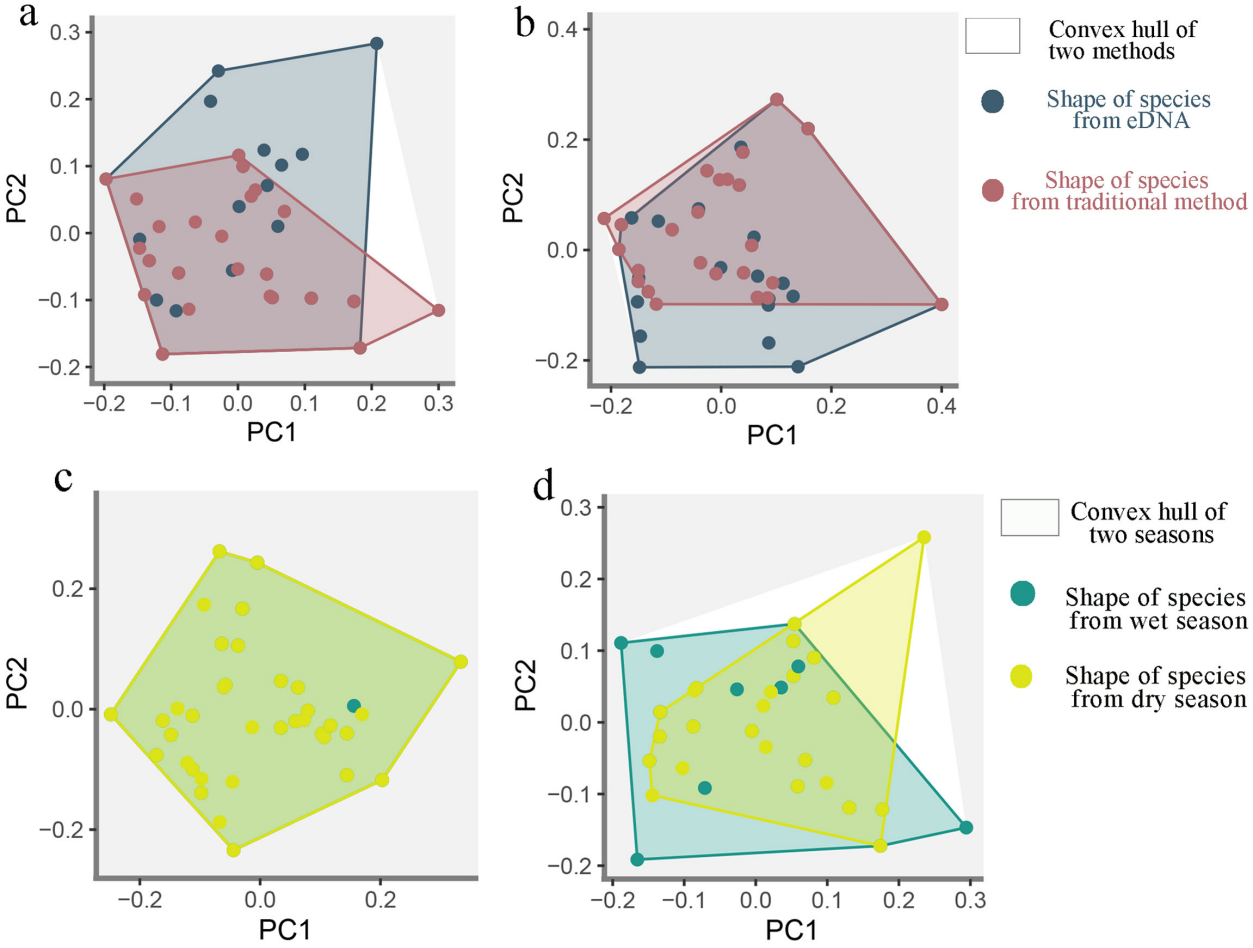
The functional spaces of the community in each season using the two methods. Differences in functional spatial composition were monitored in the wet (a) and dry seasons (b). Functional space composition difference between seasons based on the eDNA metabarcoding (c) and traditional method (d).

The results of functional traits analysis revealed that eDNA‐based results covered a larger area in the functional space compared with traditional‐based results in the dry and wet seasons. The functional richness of eDNA‐ and traditional‐based results was 0.966 and 0.450 in the dry season and 0.884 and 0.251 in the wet season (Figure 4a,b). These findings suggest that eDNA‐based results detected more functional entities compared with traditional results. The eDNA results suggest that the functional richness was usually consistent between the dry (0.998) and wet seasons (0.938), with the minor difference attributed to the absence of *Culter alburnus* in the wet season (Figure 4c). Nevertheless, the traditional survey method revealed differences in functional space and functional richness between the dry season (0.546) and the wet season (0.451). For example, *Acheilognathus macropterus* was detected only in the dry season, thereby contributing to the differences in fish community functional space between the seasons (Figure 4d).

Null model analysis showed that the trait assembly patterns of fish communities were consistent in the dry and wet seasons (Table 1). SES FEve and SES FDiv showed evidence of trait under‐dispersion based on traditional surveys during the dry season and the wet season. SES FDiv demonstrated trait under‐dispersion based on eDNA metabarcoding in both seasons.

**TABLE 1:** Results of one‐tailed test of standard effect sizes (SES) based on the null model for each of the functional diversity indices.

	SES FRic	*p*	SES FEve	*p*	SES FDiv	*p*
*Dry season*						
Traditional	−0.246	0.274	**−1.781**	**0.032**	**−2.253**	**0.001**
eDNA	0.529	0.313	−1.515	0.079	**−1.321**	**0.047**
*Wet season*						
Traditional	−0.354	0.223	**−1.895**	**0.026**	−1.230	0.105
eDNA	0.177	0.454	0.237	0.313	**−1.921**	**0.028**

*Note:* SES and *p* values are given. Significant results are presented in bold. Negative/positive SES values represent under/overdispersion of trait distribution compared to random expectation. “SES FRic”: standard effect sizes of functional richness;“SES FEve”: standard effect sizes of functional evenness; “SES FDiv”: standard effect sizes of functional divergence.

**FIGURE 5:**
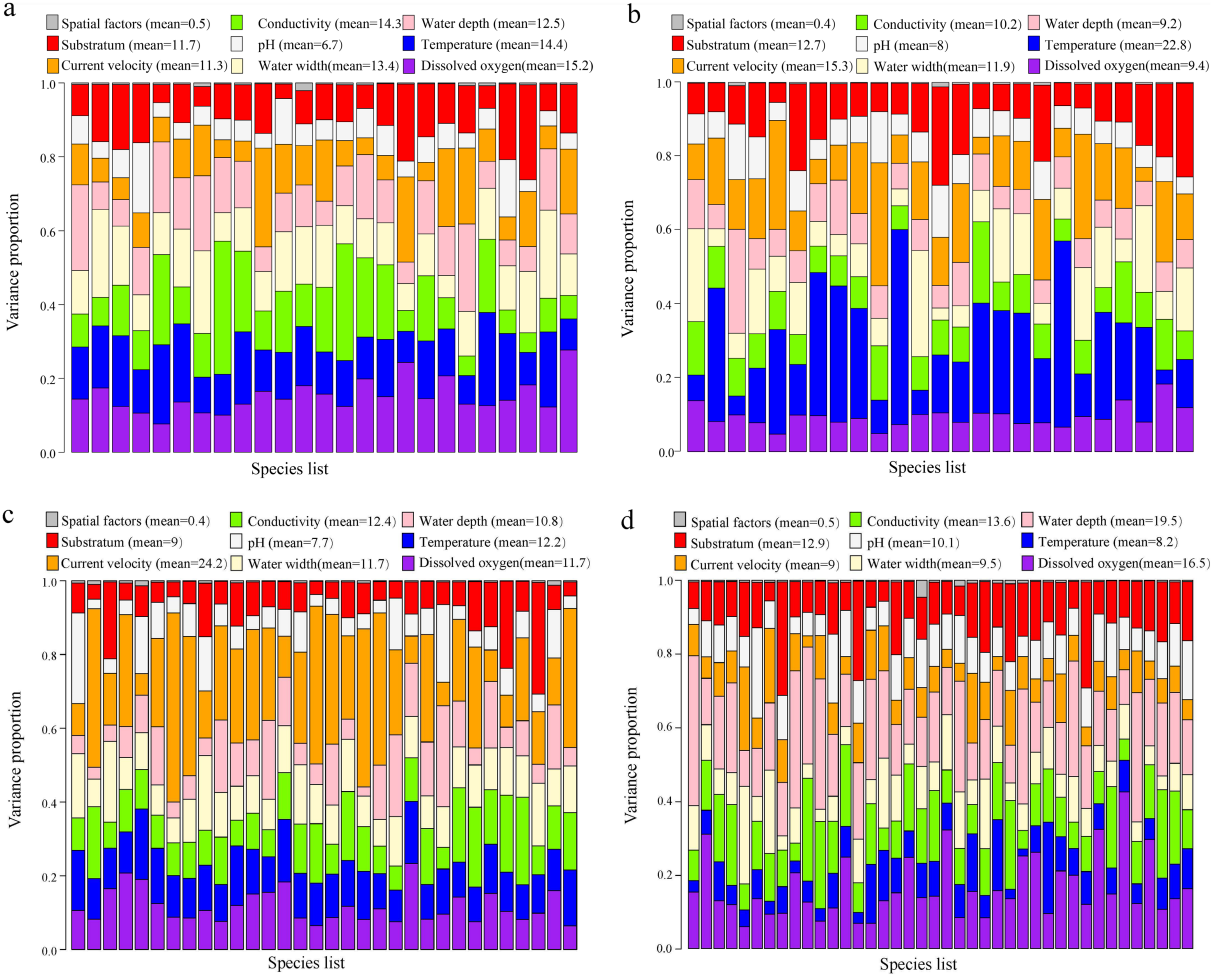
Variance partitioning of fish communities based on results of the two methods in different seasons. Each bar corresponds to a species, and various colors represent the proportion of variation elucidated by distinct environmental factors and spatial effects. Based on the traditional method in wet (a) and dry (b) seasons. Based on the eDNA metabarcoding in wet (c) and dry (d) seasons.

In the HMSC models, the AUC values exceeded 0.99, indicating outstanding performance for all fitted models. Variance partitioning based on traditional data and eDNA data revealed that environmental filtering emerged as the predominant process driving fish community assembly throughout both seasons. Specifically, in the wet season, traditional‐based and eDNA‐based results showed that environmental factors explained 99.5% and 99.6% of the variation in the fish community, respectively. Similarly, in the dry season, traditional‐based and eDNA‐based environmental contributions were 99.6% and 99.5%, respectively (Figure 5). These findings collectively suggest minimal effects of spatial factors (< 1%) on the fish community in the Huishui stream. However, the specific environmental factors dominating fish community assembly differed between seasons and survey methods. In the wet season, dissolved oxygen emerged as the most dominant factor, accounting for 15.2% of the variation, whereas temperature dominated in the dry season, explaining 22.8% of the variation (Figure 5a,b). Conversely, for the eDNA survey method, current velocity was the dominant factor, contributing to 24.2% of the variation in the wet season, whereas water depth (19.5%) was the dominant factor in the dry season (Figure 5c,d).

## 4 Discussion

eDNA metabarcoding has garnered increasing attention in the studies on fish populations and community dynamics in freshwater ecosystems. However, elucidating fish community assembly mechanisms based on eDNA often lacks validation from traditional methods. In this study, we integrated traditional survey methods with eDNA metabarcoding to determine fish community structure and assembly mechanisms in a subtropical stream. Although the taxonomic and functional structures of fish communities observed through eDNA metabarcoding and traditional methods differed to some extent, the inferred community assembly processes from survey methods were relatively consistent.

### 4.1 Fish Species Detected by the Two Survey Methods

Compared with traditional survey methods, eDNA metabarcoding usually shows similar or superior biomonitoring efficiency and fish species richness (Valentini, Pompanon, and Taberlet 2009; Yao et al. 2022). Some studies even indicate that the outcomes of a single eDNA biomonitoring session resemble those of cumulative traditional biomonitoring sessions (Cantera et al. 2019; Nakagawa et al. 2018). In this study, the species richness detected by eDNA metabarcoding surpassed that of the traditional method in the wet and dry seasons. In addition, eDNA metabarcoding detected several alien species (e.g., *Oreochromis niloticus* ) and native invasive species (e.g., *Culter mongolicus*) (Scott and Helfman 2001), which had not been previously identified in the historical survey of the Huishui stream using traditional methods (unpublished data). These species are challenging to capture via traditional methods due to their low individual density. However, the detection of these invasive species through eDNA metabarcoding can be attributed to false‐positive errors (Guillera‐Arroita et al. 2017) because human activities cannot be entirely ignored (e.g., *O. niloticus* is a common aquatic food). Conversely, false negative results may occur because eDNA signals are influenced by abiotic and biotic factors (Wang et al. 2021). The production of fish eDNA can increase with individual mass, and eDNA released by demersal fish may concentrate in sediments rather than surface water (Sakata et al. 2020; Yates et al. 2021). In the Huishui stream, many small and demersal fish species captured by electrofishing, namely *Cobitis sinensis* and *Sarcocheilichthys Parvus* , were not detected by eDNA metabarcoding in the two seasons.

### 4.2 Seasonal Variation in Taxonomic and Functional Structure of the Fish Communities

The seasonal dynamics of fish community composition are usually attributed to differences in environmental factors and fish dispersal processes (Erős et al. 2014; Fernandes et al. 2014). The effectiveness of eDNA metabarcoding in cross‐seasonal studies is influenced by many factors, such as eDNA release and persistence in the water body (Barnes et al. 2014; Rourke et al. 2022). For instance, fish typically release more eDNA into the water body during spawning seasons. Conversely, high water temperatures can increase eDNA decomposition, affecting biomonitoring results (Lamb et al. 2022; Wu et al. 2022). In the present study, more species were detected during the dry season using eDNA metabarcoding, possibly due to higher water temperatures and faster flow rates, increasing eDNA degradation in the wet season (Curtis et al. 2021; Eichmiller, Best, and Sorensen 2016). eDNA and traditional methods can detect seasonal differences in the taxonomic structure of fish communities, due to hydrological changes and associated species dispersal processes. This implies that eDNA signals have the potential to integrate seasonal shifts in fish taxa on a basin scale (Milhau et al. 2021).

Compared with taxonomic composition, eDNA metabarcoding offers greater certainty in estimating the functional structure of fish communities (Aglieri et al. 2021; Condachou et al. 2023). The assessment of functional structure from eDNA metabarcoding relies on species‐level trait datasets (Condachou et al. 2023). We observed higher functional richness and a larger functional space based on eDNA metabarcoding compared with the traditional method, which is attributed to the detection of more fish species by eDNA metabarcoding in the two seasons. Species with small populations but specialized traits may have significant effects on the evaluation of functional structure (Leitão et al. 2016). In this study, based on the traditional method, *Acheilognathus macropterus* was detected in the dry season but not in the wet season using the traditional method, leading to differences in the functional space of fish communities between seasons (Figure 4d). The functional richness and space based on eDNA metabarcoding were consistent in the two seasons. These findings indicate that eDNA metabarcoding is highly robust and comprehensive in estimating functional richness (Marques et al. 2021). However, we did not include negative controls in this study and were unable to completely exclude the effects of sequencing artifacts, such as tag‐jumping, which may affect the judgment of false‐positive errors to some extent (Schnell, Bohmann, and Gilbert 2015; Rodriguez‐Martinez et al. 2023). This also reflects the potential biases of eDNA monitoring—false‐positive errors may homogenize variance in biological communities affect the monitoring results of multi‐community (Yao et al. 2022).

### 4.3 The Driving Factors of the Community Assembly Mechanism

The results of null model analysis showed that the fish communities detected by the two survey methods showed trait under‐dispersion patterns, indicating that the fish communities were structured by environmental filtering during the dry and wet seasons (Mori et al. 2015). Although eDNA detected more functional entities compared with traditional methods, the trait patterns consistent with traditional methods indicated that these additional species were also screened by the upstream habitat, reflecting the effectiveness and robustness of eDNA in inferring trait‐based community assembly (Marques et al. 2021; Condachou et al. 2023). However, this is based on a clear understanding of the species and trait background of the study area; otherwise, eDNA‐based methods may be limited, for example, in tropical areas where species are rich but genetic data are relatively scarce (Marques et al. 2021).

Consistent with the null model inference, we determined that both eDNA metabarcoding and electrofishing results highlighted environmental filtering as the dominant process shaping fish communities in the Huishui stream across two seasons using the HMSC framework. According to the network position hypothesis, community assembly in upper streams is primarily influenced by environmental factors rather than spatial effects due to high environmental heterogeneity and isolation within the river network (Brown and Swan 2010). These findings are consistent with general expectations for fish communities in the upper reaches of subtropical rivers (Carvalho et al. 2023; Vieira and Tejerina‐Garro 2020; Yang et al. 2023), highlighting the reliability of eDNA metabarcoding in inferring community assembly processes. Altogether, although minor discrepancies in biomonitoring results between the two survey methods exist, they do not undermine the credibility of eDNA metabarcoding in inferring the assembly mechanisms of upstream fish communities, consistent with the findings of Hallam et al. (2021) in their study of fish metacommunity based on eDNA and traditional methods.

## 5 Conclusion

Our study reveals that, in comparison to the traditional survey method (electrofishing), the results obtained from eDNA metabarcoding are comparable, if not superior, in assessing the taxonomic and functional structure of fish communities in upstream reaches. Despite significant differences in fish taxonomic composition between the two seasons, the consistency in community assembly mechanisms across seasons highlights the importance of exploring the connections between functional structure and community assembly in eDNA‐based research. Drawing from our investigation into the assembly mechanisms of fish communities in the upper reaches of a subtropical stream, we conclude that eDNA can reliably infer fish community assembly across seasons.

## Author Contributions

**Ziyu Liu:** investigation (equal), software (equal), writing – original draft (equal). **Cong Hu:** data curation (equal), software (equal). **Wenhui You:** investigation (equal). **Shuxin Li:** investigation (equal). **Yongsheng Wu:** data curation (equal). **Yangyang Liang:** supervision (equal). **Ling Chu:** supervision (equal). **Yunzhi Yan:** conceptualization (equal), methodology (equal). **Chen Zhang:** funding acquisition (equal), supervision (equal), writing – review and editing (equal).

## Conflicts of Interest

The authors declare no conflicts of interest.

## Supporting information


Appendix S1.

## Data Availability

The original data are deposited on figshare. https://figshare.com/s/db8cd2aecd8aff01ae2c.
