# Supplementary material for: Comparison Between Environmental DNA Metabarcoding and Traditional Survey Method to Identify Community Composition and Assembly of Stream Fish
Source: Ecol Evol. 2024 Nov 25;14(11):e70627. doi: 10.1002/ece3.70627 (PMC11588354; doi:10.1002/ece3.70627)
Supplement: Supplementary file 1 — Appendix S1. [file ECE3-14-e70627-s001.docx]

**Table S1 Fish morphological features and functional traits**

| **Functional traits** | **Ratio** | **Ecological significance** | **References** |
| --- | --- | --- | --- |
| Relative eye size | $\frac{ED}{HD}$ | Visual scope to food | Boyle&Horn，2006 |
| Mouth gape position | $\frac{MH}{HD}$ | Living position in water | Sibbing&Nagelkerke，2000 |
| Relative gut length | $\frac{GL}{SL}$ | Ability to digest food | Kramer&Bryant，1995 |
| Eye position | $\frac{EH}{HD}$ | Vertical position in water | Gatz，1979 |
| Body shape | $\frac{BD}{BW}$ | Habitat location  and swimming ability | Sibbing&Nagelkerke，2000 |
| Body height | $\frac{BD}{SL}$ | Habitat location  and swimming ability | Sibbing&Nagelkerke，2000 |
| The contribution of Caudal peduncle to swimming | $\frac{CFH}{CPH}$ | Swimming endurance | Webb，1984 |

*SL*： Standard length； *BW*： Body width； *BD*： Body depth； *HD*： Head depth；

*MH*： Mouth height； *GL*： Gut length； *EH*： Eye height； *CFH*： Caudal fin

height； *CPH*： Caudal peduncle height.
